# Current Translational 3D In Vitro Models of Human Placental Tissue

**DOI:** 10.1007/s12015-026-11231-4

**Published:** 2026-09-12

**Authors:** Ananya Raman, Niamh E. P. Mills, Louise Cole, Claire Richards, Lana McClements

**Affiliations:** 1 School of Life Sciences, Faculty of Science, University of Technology Sydney, Sydney, NSW Australia; 2 Institute for Biomedical Materials and Devices, Faculty of Science, University of Technology Sydney, Sydney, NSW Australia

**Keywords:** Placenta, Pregnancy, Trophoblast cells, Organoids, Placenta-on-a-chip, Bioprinting

## Abstract

Cellular models of placental tissue have the potential to recapitulate normal physiology, model disease, and facilitate therapeutic drug testing. In vivo or two-dimensional (2D) in vitro models of placental conditions have not been sufficiently representative of whole human tissue, thus have limited physiological relevance. This can be attributed to interspecies differences in placental physiology or a lack of structural complexity. In comparison, three-dimensional (3D) multicellular model systems provide a more sophisticated analogue of human placental tissue. Newer models of human placenta include 3D organoids, placenta-on-a-chip and organoid-on-a-chip, employing a range of materials and methods including pluripotent or trophoblast stem cells, microfluidics and 3D bioprinting. Accurately mimicking in vivo human tissue is crucial to the success of these translational 3D models of the placenta.

In turn, a reliable representation of placental tissue architecture will allow discovery of the mechanisms underlying abnormal placental development and inform the prevention, diagnosis and treatment of pregnancy-related disorders such as preeclampsia and fetal growth restriction. Here, we provide a comprehensive critical appraisal of recent advances in 3D placental models, which aim to elucidate placental physiology, development and dysfunction. Additionally, we discuss how these platforms can accelerate the development of better monitoring and treatment strategies for pregnancy complications induced by a dysfunctional placenta.

**Graphical Abstract:**

**Figure:**
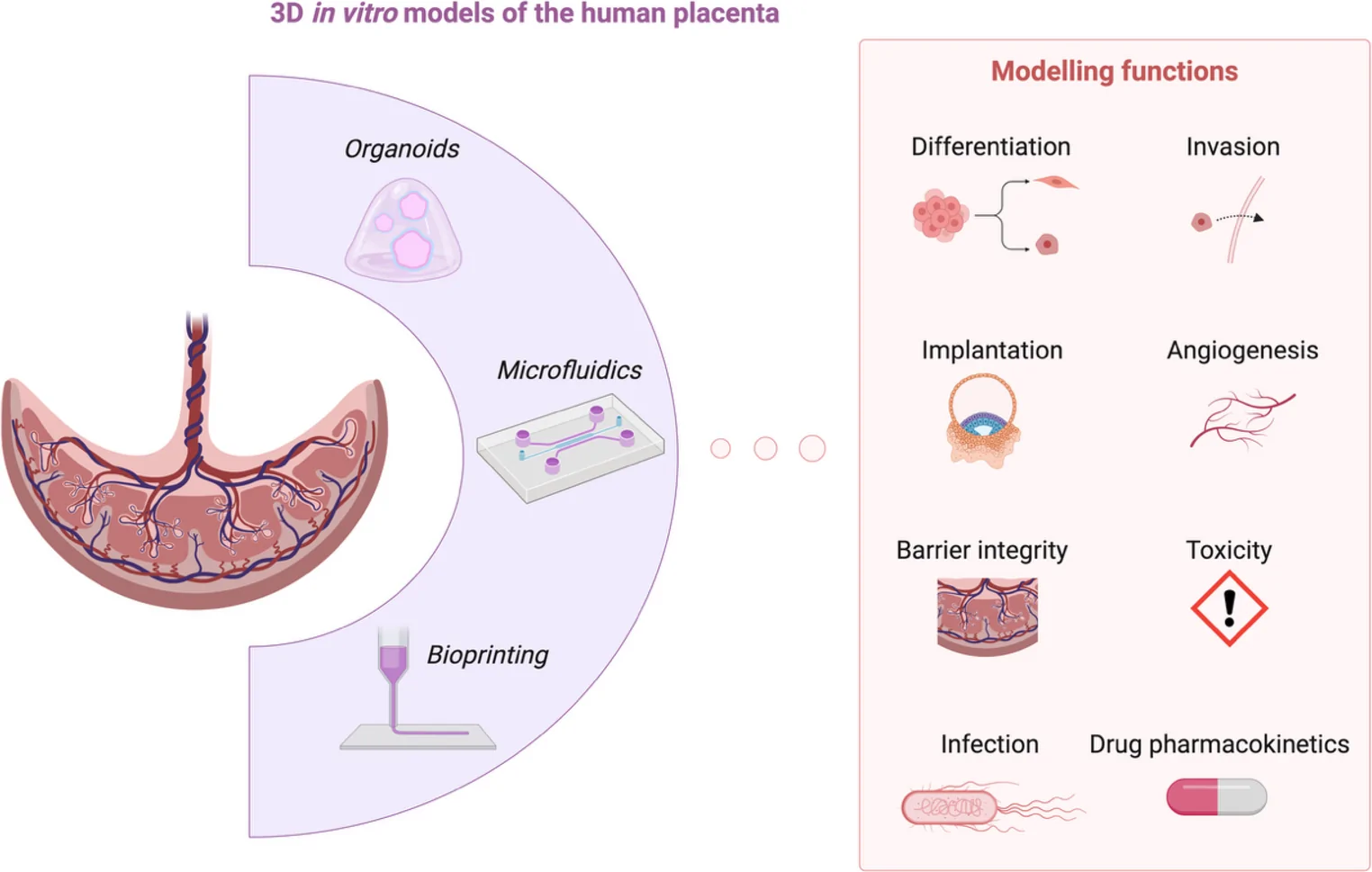
Processes occurring during early pregnancy remain poorly understood, necessitating the use of authentic models. Researchers have set out to recapitulate placental cell interactions in 3D cell models that more closely reflect the tissue of origin

## Introduction

The placenta is an extra-embryonic organ that forms during pregnancy and, albeit temporary, it is critical for fetal development and growth [1–3]. We know that cellular and immunological imbalances during human placental development can result in adverse pregnancy outcomes including preeclampsia, fetal growth restriction, spontaneous abortion, infection and pre-term birth [4, 5]. Many of the biological processes that underpin these conditions originate during early pregnancy, encompassing the peri-implantation period and first trimester, when critical events such as endometrial decidualisation, embryo implantation, trophoblast differentiation and invasion, and placental establishment occur. Defects in these tightly regulated processes can lead to abnormal placentation and are thought to contribute to the later manifestation of pregnancy complications [6–8]. Yet processes occurring during early pregnancy remain poorly understood due to difficulty in safely and ethically obtaining samples during pregnancy [9]. This limited access has hindered the identification of reliable early predictors of disease and restricted understanding of the mechanisms driving placental dysfunction and subsequent pregnancy disorders. Consequently, physiologically relevant models that reflect early placental development and function are essential for investigating disease pathogenesis, identifying predictive biomarkers, and evaluating potential therapeutic interventions.

Given the limited accessibility of early human pregnancy tissue, researchers rely on experimental models to investigate placental development and dysfunction, including in vivo animal models and in vitro cell culture systems. In many cases, in vivo animal models are used to study organ interactions within an entire biological system. Such animal models have helped elucidate placental physiology and development, though their ability to simulate aberrant placentation that occurs during preeclampsia in humans has been questioned [10–12]. Mice are popular model systems for human pregnancy because, like humans and simian primates, they have a haemochorial placenta wherein syncytiotrophoblasts are in direct contact with the maternal blood [11, 13]. However, placental development is highly divergent between species. For example, normal pregnancy in mice involves shallow trophoblast invasion. In comparison, this phenotype would indicate placental dysfunction in humans, given that deep trophoblast invasion through the decidua and the inner myometrium is a key feature of human pregnancy. This process has only been observed at similar depth and routes in certain species within the Hominidae (great ape) family, which is stipulated to have originated in the last common ancestor of humans, gorillas and chimpanzees [14]. Additionally, complications such as gestational hypertension and preeclampsia have only been observed spontaneously in humans and, rarely, in certain non-human primates [10, 12]. Furthermore, the human placenta has a molecular profile distinct from primate or murine placentae, including expression of chorionic gonadotropin in the trophoblast lineage [11]. These differences in pregnancy between species necessitate inducing preeclampsia in animal models through immunological, pharmacological, genetic, or surgical methods [12]. Therefore, the ability of animal models to provide accurate insights into human placentation is limited.

While animal models enable investigation of complex physiological interactions within a whole organism, cell culture models provide controlled platforms for studying human-specific cellular mechanisms. Conventional 2D in vitro cell culture models where cells are grown as monolayers on rigid culture surfaces are simpler, more cost-effective and a well-established methodology compared to 3D models [15, 16]. In vivo, cells exist within a complex 3D microenvironment composed of neighbouring cells, extracellular matrix (ECM) proteins and signalling molecules. 2D models cannot fully reproduce this microenvironment because cell-cell and cell-matrix interactions remain simplified relative to native tissue, limiting their ability to recapitulate physiological tissue architecture and function. As a result, these systems often fail to capture the complexity and heterogeneity of placental tissue. Collectively, these limitations have driven the development of more physiologically representative preclinical models of placental tissue.

**Fig. 1:**
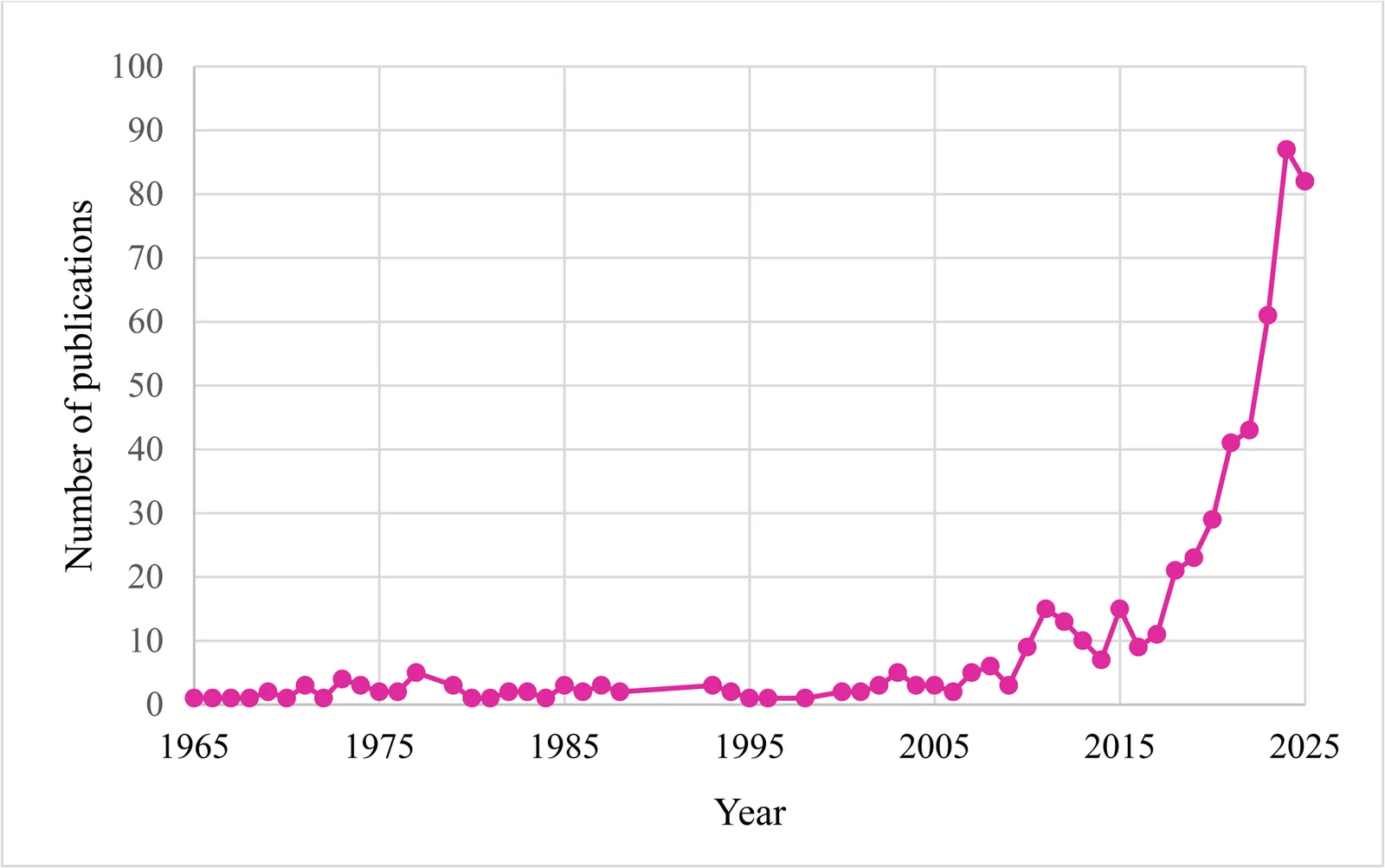
Number of placental spheroid and organoid research articles published per year. A PubMed search was conducted for papers with (‘placenta’ or ‘trophoblast’) AND (‘spheroid’ or ‘organoid’) NOT ‘review’. Resulting publication numbers are graphed per year up to 31st December 2025

**Fig. 2:**
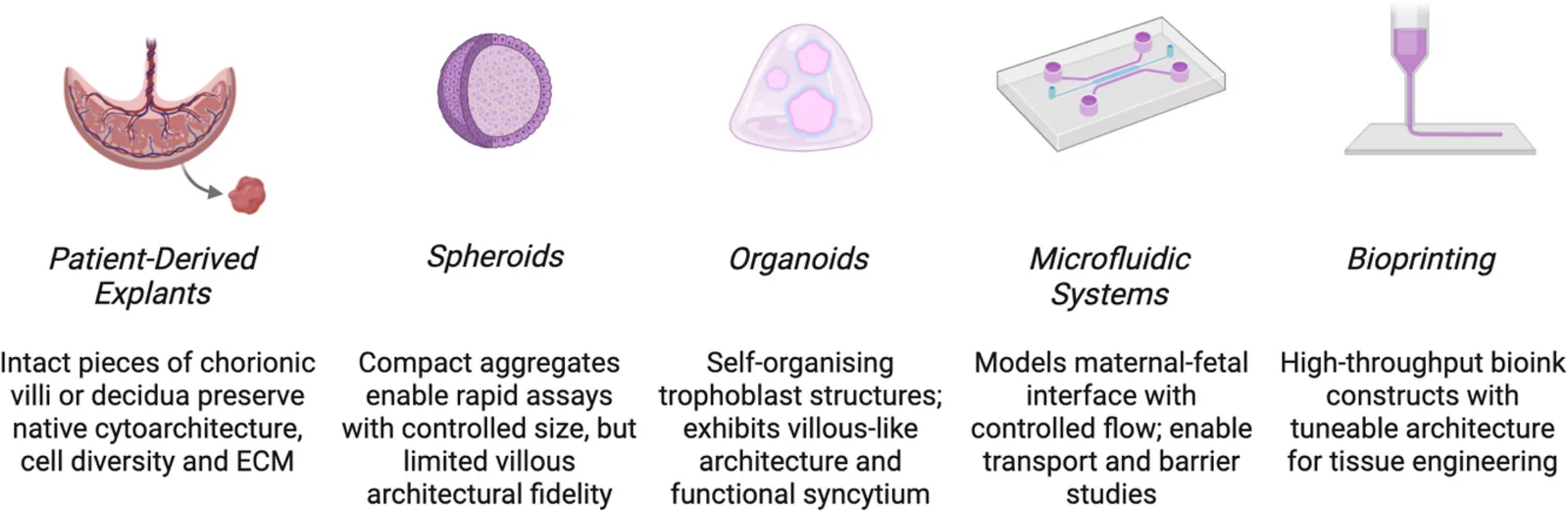
Comparison of 3D in vitro models of placental tissue. Patient-derived explants comprise intact pieces of chorionic villi or decidua. They are a mainstay of in vitro placental research and preserve the native placental microarchitecture, cell diversity and ECM. Newer in vitro models include spheroids, organoids, microfluidic systems such as placenta-on-a-chip, and high-throughput technologies like bioprinting

Continued efforts to improve physiological relevance evolved cell culture systems from conventional 2D monolayers to increasingly sophisticated 3D cell models that more closely reflect the tissue of origin, often with structural support provided by ECM components [15, 17]. When reviewing the literature on placental spheroid and organoid cultures, it is apparent that their use has increased dramatically in the last decade, facilitating research and a better understanding of placental physiology and pathologies (Fig. 1). Such models include 3D trophoblast organoids established from first-trimester human placentae that mimic trophoblast complexity, endocrine activities and metabolic processes [18, 19]. This has progressed further into more complex 3D cell culture models using technology such as microfluidic devices and bioprinting to model cell migration and invasion, cell-cell interactions and cell-environment interactions (Fig. 2).

A reliable model of the placenta must recapitulate placental cellular organisation, tissue function and morphology, as well as metabolic and endocrine activity, while also capable of reflecting the heterogeneity of placental insufficiency [1]. Another important consideration is the cell type used in 3D cell culture models. For example, some trophoblast cell lines (e.g. BeWo, JEG-3, JAR) are of choriocarcinoma origin enabling multiple passages, though their genetic signatures may differ from primary trophoblasts. Primary trophoblast cells are isolated directly from the placenta and are therefore considered more representative, nevertheless, they are challenging to obtain. In addition, although they offer greater phenotypic and genomic/proteomic stability over cell lines, primary cells cannot be cultured for an extended period as they differentiate and initiate apoptosis. The limited lifespan of primary first-trimester trophoblast cultures is improved by establishing organoids [20, 21].

This paper aims to review advances in 3D placental models that accurately encompass physiological and dysfunctional placental conditions to help develop reliable diagnostics, treatments and monitoring strategies.

## Placental Cell Types and their Functions

The placenta is the first organ formed during embryogenesis and plays a vital role during its transient existence [22]. It is firmly embedded in the decidua, forming the maternal-fetal interface and structurally connects the developing embryo with the mother’s body. It is also highly functional and complex; it transports nutrients necessary for fetal development, exchanges oxygen and carbon dioxide, removes waste, and protects the fetus from maternal antigens which could pose an immunological risk.

Placental development starts soon after conception, when the fertilised zygote develops and divides into a blastocyst with an outer layer of trophoblast stem cells (TSCs) known as the trophectoderm [23–25]. As the blastocyst embeds itself within the decidualised endometrium, the trophectoderm gives rise to pluripotent, progenitor cells known as cytotrophoblasts (CTBs) that start to form the villous structures of the placenta [23]. These CTBs can self-renew, fuse to form the multinucleated syncytiotrophoblast (STB) layer or differentiate into highly invasive extravillous trophoblasts (EVTs). The STB layer forms a barrier between fetal and maternal circulation while permitting selective diffusion of molecules between [23, 26]. EVT subtypes, including interstitial EVTs and endovascular/endoarterial EVTs play a critical role in invading the decidua and remodelling the spiral uterine arteries up to the inner third of the myometrium to form low-resistance vessels [23, 27]. This is a key event in early pregnancy which allows for adequate placental perfusion throughout gestation.

The placenta comprises an extensive vascular network, culminating within the placental villi [22]. Vascular function in the human placenta is maintained by projecting chorionic villi, which separate maternal blood from the fetal circulation while distributing blood to the developing fetus. Fetal vascular cells include endothelial cells and mural cells, comprising vascular smooth muscle cells and pericytes. Placental microvascular endothelial cell function is highly influential on the success of a pregnancy. Their angiogenic activity guides the rapid expansion of the placenta in the first and second trimesters, and the halt in growth as the pregnancy approaches to term. On the maternal side, vascular smooth muscle cells provide structural support to placental blood vessels and regulate their dilation, which allows for greater volumes of maternal blood to reach the intervillous space. Pericytes surround endothelial cells, support endothelial cell function while restricting their migration and proliferation [28]. This helps maintain microvessel stability and integrity, particularly in the terminal villi, while regulating blood supply and flow.

Adequate maternal-fetal circulation is crucial for fetal homeostasis; as a result, placental vasculopathies compromise both maternal and fetal health [22]. Features such as altered angiogenic factors, endothelial dysfunction and reduced vascular perfusion have been linked to conditions such as preeclampsia, fetal growth restriction and early pregnancy loss [2]. Therefore, it is essential to incorporate vascularity into 3D placental models.

**Fig. 3:**
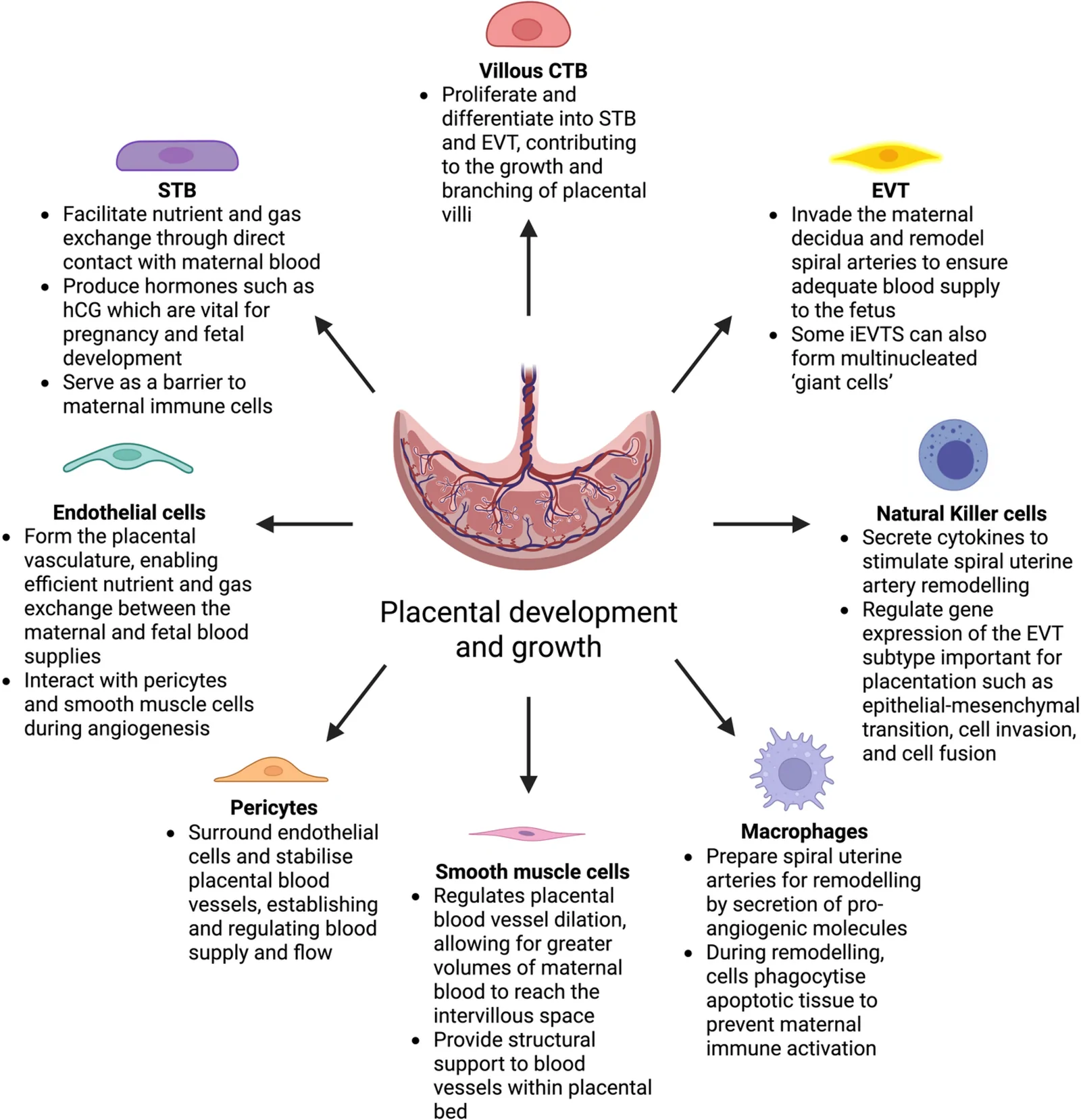
Summary of the different cell types and their roles in placental development and growth. Various cell types perform key functions during placentation. These include trophoblast cells (the undifferentiated progenitor, villous cytotrophoblast, and differentiated extravillous and syncytiotrophoblast lineages), immune cells such as uterine natural killer cells and decidual macrophages, and cell types comprising blood vessels (endothelial and mural cells). CTB, cytotrophoblast; EVT, extravillous trophoblast; hCG, human chorionic gonadotropin; iEVT, interstitial EVT; STB, syncytiotrophoblast. Figure created in BioRender.com

Throughout pregnancy, the maternal immune system plays an essential role in placental development, protecting the fetus from pathogens, and inducing tolerance to the semi-allogenic fetus [29]. An intricate balance between innate immune cells and placental/uterine cell interactions and functions is necessary to achieve this. At the maternal-fetal interface, a complex network of cellular connections is established. Immune cells such as natural killer (NK) cells, macrophages, T cells, dendritic cells and B cells interact with decidual stromal cells (DSCs) including mesenchymal stem/stromal cells or fibroblasts, smooth muscle cells and trophoblasts [30]. Uterine NK (uNK) cells and macrophages prepare the spiral uterine arteries for remodelling and EVT invasion [29, 31]. uNK cells and macrophages in endometrial tissue release placental growth factor and vascular endothelial growth factor, a process necessary for vascular remodelling and angiogenesis [32–34]. Decidual macrophages also prevent the release of self-antigens or paternal alloantigens to maintain tolerance to the fetus [35]. Fetal-origin placental macrophages known as Hofbauer cells (HBCs) are found in the chorionic villi and are one of the most abundant immune cells in the human placenta, yet their role in fetal protection and tolerance is not well-characterised [36–38]. In healthy pregnancies, HBCs generally polarise into the M2 phenotype which secretes anti-inflammatory cytokines and promotes tissue repair and remodelling. HBCs also play a role in regulating placental angiogenesis and vasculogenesis, villous trophoblast differentiation, and the absorption of maternal immune complexes [39]. Overall, these different cell types facilitate key processes during placentation such as rapid placental expansion, trophoblast invasion and spiral artery remodelling through extensive biochemical and cellular crosstalk (Fig. 3). These interactions are mediated through direct cell-cell contact and the exchange of signalling molecules within the placental microenvironment [40, 41]. Therefore, 3D co-culture models that incorporate multiple placental, vascular and immune cell populations are likely to more faithfully recapitulate human placental physiology and pathology than models consisting of a single cell type.

## Current 3D Models and Studies of the Human Placenta

### Patient-Derived Explants

Prior to more recent advances in in vitro techniques such as spheroids and organoids, explants were one of the mainstays of placental research [9]. Patient-derived placental explants are cultured directly from dissected primary placental tissue. These small, intact pieces preserve the cytoarchitecture, cell diversity and matrix of the native tissue. In culture, explanted first-trimester villi show trophoblast outgrowth and migration into ECMs such as Matrigel® (Corning) [42, 43]. After an initial adaptation period, human placental explants regain structural and functional properties by around 48 hours and can maintain overall tissue viability and endocrine function for approximately 7–11 days [44, 45]. The main strength of these models is their relevance to native human placenta, while still allowing for experimental manipulations. However, explant culture is constrained to a limited lifespan, with loss of viability of the mesenchymal core after approximately 48 hours, and differential survival of specific cell types over time without perfusion [9, 44].

### Organoids and Spheroids

Organoids are self-organising, multicellular constructs arising from individual stem cells that mimic functional, structural and biological aspects of an organ [46]. This contrasts with spheroids, which are 3D cell aggregates whose organisation primarily arises through cell-cell aggregation and adhesion. While spheroids can reproduce certain aspects of 3D cell-cell interactions, they generally lack the spatial organisation, cellular heterogeneity and tissue architecture observed in native tissues. In contrast, organoids undergo both self-assembly and self-organisation, whereby cells differentiate in response to physical and chemical cues to generate multiple cell types arranged into more complex, physiologically relevant structures [46]. Consequently, organoids more closely resemble native tissue architecture, cellular heterogeneity and function.

#### Organoid Microenvironment

**Fig. 4:**
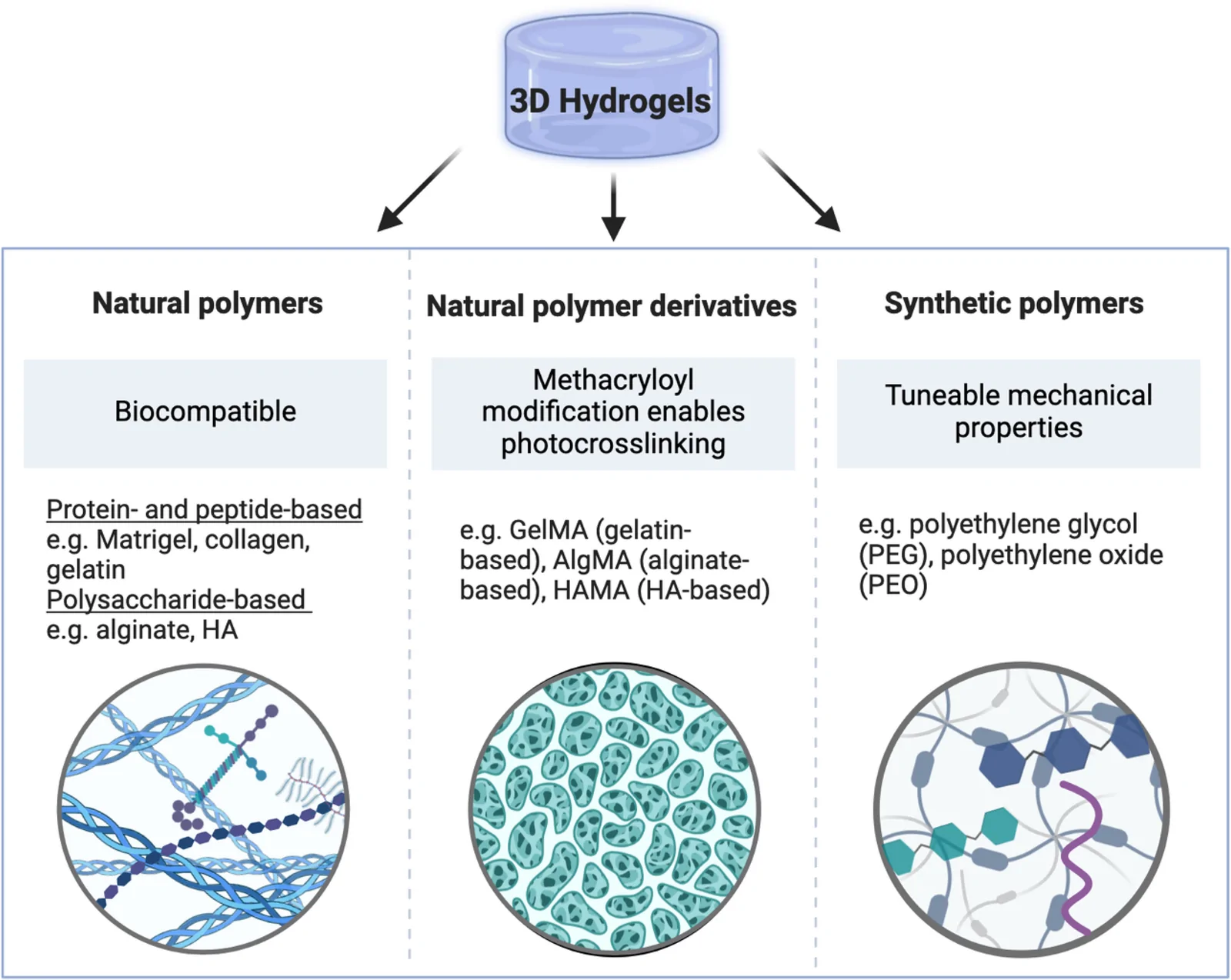
Extracellular matrix polymers used in the construction of 3D hydrogels. Natural polymers have the advantage of biocompatibility. These include protein- and peptide-based biomaterials such as collagen and gelatin, and polysaccharide-based biomaterials such as alginate and hyaluronic acid (HA). Derivatives of natural polymers include gelatin methacryloyl (GelMA), alginate methacryloyl (AlgMA) and hyaluronic acid methacryloyl (HAMA), all of which are modified with the photosensitive group methacryloyl to enable light-based crosslinking. Synthetic polymers include polyethylene glycol (PEG) and polyethylene oxide (PEO). These biomaterials allow tuning of mechanical properties such as stiffness, elasticity and porosity. Figure created in BioRender.com

Hydrogels are complex polymer networks that form a scaffold, support cellular growth, and allow manipulation of microenvironmental properties to better replicate the tissue of interest and its native ECM (Fig. 4). Organoids are typically established by embedding stem cells within hydrogels containing polymers that mimic aspects of the in vivo microenvironment, supporting cellular proliferation and differentiation [1]. Beyond their structural role, ECM components actively regulate cell behaviour through biochemical and biomechanical signalling. Matrix properties such as composition, stiffness, elasticity, porosity, degradability and conductivity can influence cellular processes including adhesion, proliferation, differentiation and migration. Consequently, selecting an appropriate hydrogel is of paramount importance [47].

Hydrogels used for organoid culture can be broadly classified as natural, synthetic or hybrid biomaterials. Natural materials include Matrigel, collagen, gelatin, fibrin, hyaluronan and elastin. These biomaterials are biocompatible and contain adhesion and degradation sites that facilitate cell-matrix interactions; however, they are often limited by their heterogeneity and lack of tuneable properties. As a result, natural polymer derivatives or synthetic hydrogel alternatives have been used as ECM biomaterials in organoids across different tissue sources [48, 49]. Hybrid biomaterials are derived from natural polymers to retain their desirable biocompatibility while allowing for the tuneability of synthetic polymers [47]. Gelatin is limited by its physical crosslinking method while the hybrid biomaterial gelatin methacryloyl (GelMA) is modified with methacryloyl groups to enable photocrosslinking. Synthetic polymers, including polyethylene glycol (PEG) and polyethylene oxide (PEO), offer greater reproducibility and tuneable mechanical properties, though they often require modification to enhance biological compatibility. The selection of a suitable hydrogel therefore requires balancing biological relevance, tuneability, reproducibility and experimental objectives.

At present, many trophoblast organoid models (and other advanced placental models, such as microfluidic chips) continue to rely on Matrigel, a cell-derived matrix. Matrigel is a basement membrane matrix extracted from Engelbreth-Holm-Swarm mouse sarcoma which contains ECM components such as laminin and collagen IV [50]. Its widespread use stems from its ease of use, accessibility and capacity to support attachment and proliferation of multiple cell types. However, proteomic analyses of Matrigel reveal significant batch-to-batch variability which can affect the reproducibility of models using this matrix [50]. While growth factor-reduced formulations are available, it is difficult to control the various components present in animal-derived biomaterials such as Matrigel, which can make it difficult to replicate tissue-specific ECM environments.

Matrices can also be functionalised with the addition of full-length proteins, enzymatically cleavable sites to create degradable hydrogels, and peptide derivatives which can be combined synergistically [51]. A major barrier to the clinical translation of these peptide-based hydrogels is expensive production costs. Despite advances, there remains a need for matrices that more accurately replicate the biochemical and biophysical properties of the native placental ECM while minimising batch-to-batch variability. Such matrices would enable precise control over key parameters, including stiffness, elasticity, conductivity and porosity, all of which influence cell behaviour, differentiation and tissue organisation.

#### Immortalised Trophoblast/Choriocarcinoma Organoids

The study of early pregnancy is complicated by difficulties obtaining human placental tissues during the first trimester. As a result, immortalised trophoblast cell lines have been used in organoid and spheroid models, many of which such as BeWo, JEG-3 and JAR originate from choriocarcinoma, allowing for longer proliferation in culture [20, 52]. There are four criteria by which Lee and colleagues defined human first-trimester post-implantation trophoblasts: the expression of protein markers cytokeratin 7, *GATA*–binding protein 3 and transcription factor activator protein-2 gamma; hypomethylation of ETS transcription factor 5 (*ELF5*) promoter; expression of human leukocyte antigen (HLA) Class I molecules; and expression of chromosome 19 microRNA cluster microRNAs [53]. Choriocarcinoma-derived cell lines have fulfilled these four criteria, although their transcriptomes can differ from primary trophoblasts [54, 55]. Immortalised primary trophoblast cells are genetically modified to overcome the limitations of carcinoma-derived trophoblast cell lines. One such cell line, HTR-8/SVneo, was established by transfecting human first-trimester primary EVTs with the gene encoding simian virus 40 large T antigen [56–59]. The resultant cell line contains a mixed population of trophoblast and stromal cells. Despite exhibiting markers characteristic of invasive EVTs in situ, HTR-8/SVneo reportedly lacks classical EVT and villous CTB markers and was unable to develop trophoblast organoids in a comparative study [52]. This has been attributed to their origination from a single trophoblast subtype, limiting their ability to reflect the physiological placenta or their capacity to form organoids. The hybrid cell line ACH-3P, on the other hand, was formed by the fusion of primary first-trimester trophoblasts with immortalised choriocarcinoma cell line, AC1-1 [21]. ACH-3P cells have been shown to contain populations of HLA-G− and HLA-G+ cells, and their transcriptome resembles that of their primary cell parents. Organoids established from ACH-3P cells have also mimicked trophoblast organoid architecture, seeded in stemness medium mixed with Growth Factor-Reduced Matrigel. These showed distinct HLA-G+ areas with subsequent EVT differentiation [52]. In 2D conditions, a functional comparison of BeWo, JEG-3, JAR and ACH-3P cells found that the most suitable cell type depended on the in vivo processes being modelled [60]. Barrier function models were best represented by ACH-3P and JEG-3 cells; nanoparticle transport was best modelled by ACH-3P; and syncytialisation was best modelled by BeWo.

Trophoblast cell lines can be more easily obtained than primary cells and are lower cost, making them an accessible, low-risk alternative cell source that can be genetically manipulated [61, 62]. In view of this, reproducible and robust 3D placental models developed using trophoblast cell lines are therefore likely to facilitate screening and drug/biomarker discovery stages before validating findings using primary cell-based models.

#### Primary Trophoblast Organoids

Placental organoids have been established from stem or progenitor trophoblast cells isolated from primary tissue, initially from the first trimester and more recently from term placentae [18, 19, 63]. The first organoids derived from primary, first-trimester trophoblast cells aimed to model the placental villi, which cannot be achieved with a 2D monolayer culture [18, 19]. In these models, primary trophoblast cells were embedded in Matrigel droplets and cultured in medium containing expansion components, growth factors and signalling inhibitors to encourage stemness and proliferation. These self-replicating human placental organoids displayed characteristics of in situ trophoblasts, such as the ability to differentiate into STBs and EVTs, with transcriptomes resembling the first-trimester placenta. However, the orientation of villous cells and STBs differed from that seen in placental tissue; the organoids had an outer villous CTB layer that could be stimulated to generate invasive EVTs, with the border STB lineage confined to the centre of the organoids. This “apical-in” structure is due to the reversed polarity of the organoids and limits the ability to which STB processes such as barrier function, shedding and regeneration can be studied. Yang and colleagues recently developed a method to address this issue by releasing their apical-in primary trophoblast organoids from the Matrigel and further culturing them, suspended in growth medium with gentle rotation [64]. Indeed, removal from the matrix was confirmed to reverse organoid polarity with the differentiation of STBs forming on the outer surface of organoids (“apical-out”), reflecting the arrangement of these cells in vivo. This maintained the organoids’ morphology and distinct trophoblast cell subpopulations, while also allowing for extended applications such as microbial infection modelling, in which large microbes are often unable to penetrate an ECM biomaterial.

As mentioned, term placenta is now also being used as a source of primary trophoblasts, given it is more widely accessible. Previous research had found that CTBs isolated in later gestation were only capable of differentiating into the STB lineage, whereas first-trimester CTBs could differentiate into both STBs and EVTs [65]. However, more recent studies have isolated side-population trophoblasts directly from third-trimester placentae using the Hoechst side-population isolation technique [66]. These side-population trophoblasts self-assembled into organoids, and had a self-renewing and differentiation capacity comparable to stem cell populations [63, 67].

In more recent years, the use of primary trophoblasts has continued to be validated in organoid models through high-resolution analysis methods such as proteomics and single-cell RNA sequencing (scRNAseq) [68, 69]. Though this also highlighted that scRNAseq has limited application to syncytialisation analyses, and techniques such as single-nucleus RNA sequencing will support a more accurate comparison of in vitro STB formation back to native tissue, as scRNAseq cannot separate out multiple nuclei sharing one common cytoplasm [70, 71].

When compared to primary placental tissue, these primary trophoblast-derived organoid models have demonstrated their ability to reflect in vivo trophoblast development and differentiation [72]. However, the widespread application of these models is hindered by challenges including limited access to primary, early pregnancy placental tissue, the extensive processes required to isolate the cell types needed, and the high costs needed to support their development. Issues around placental tissue availability and ethical restrictions have prompted the use of human pluripotent stem cells (hPSCs) and human trophoblast stem cells (hTSCs) in research to model human trophoblast differentiation.

#### Native and Induced Trophoblast Stem Cell Organoids

TSCs and hPSCs are increasingly used in multicellular models to simulate trophoblast differentiation while also addressing barriers to primary tissue access. A study by Zdravkovic and colleagues successfully derived human embryonic stem cells (hESCs) from single blastomeres to create a CTB progenitor cell line with TSC potential [73]. However, the first bona fide TSC cell line derived directly from primary tissue was cultured by Okae and colleagues from blastocysts and first-trimester human placenta (6–9 weeks gestational age) [74]. These ‘Okae TSCs’ were capable of long-term maintenance in culture and demonstrated a capacity for lineage-specific differentiation into both STBs and EVTs, with transcriptome profiles indicating key markers for human trophoblast cells. Gene expression analyses showed that these stem cell lines were most like peri-implantation trophoblasts. They were also highly proliferative and could be cultured long-term or cryopreserved. As such, these cells have allowed for trophoblast characterisation in early placental development and the study of disorders arising from trophoblast dysfunction. In short, the development of these hTSCs represented a significant breakthrough in the placental and trophoblast research community since the TSCs and differentiation protocols have now been and continue to be used by many research groups worldwide [18, 75–79].

Despite this breakthrough, the use of pre-implantation embryos and early-gestation placenta, as described by Okae and colleagues, remains challenging due to unknown pregnancy outcomes, costs, and ethical concerns with the use of pre-implantation embryos and early first-trimester placentae from pregnancy terminations [79]. More recently published protocols aim to address these challenges using the Okae et al. method to derive TSCs from hPSCs, which include induced pluripotent stem cells (reprogrammed from somatic cells) and hESCs (derived from the human embryo) [65]. These new protocols have included the use of both naïve (pre-implantation) and primed (post-implantation) stem cells.

Further studies by Okae and colleagues established a conversion system wherein naive hESCs transdifferentiated into both EVT-like and STB-like hTSCs [80]. This study also compared naïve and primed hESCs but found that the proliferation and differentiation of the primed cells was inferior. In contrast, primed hPSCs were considered to generate “bona fide” TSCs in another study, based on criteria for trophoblast identity including the presence of a trophoblast gene signature and *ELF5* promoter hypomethylation [77]. Comparative analysis of a study by Viukov and colleagues demonstrated that hPSC-derived TSCs were able to self-renew or differentiate into main trophoblast lineages due to residual plasticity, and had RNA and epigenetic profiles equivalent to previous TSC lines derived from placenta or naïve PSCs [81]. TSCs have also been derived through directly reprogramming dermal fibroblasts using TSC culture conditions and Yamanaka pluripotency factors [82, 83]. Thus, the establishment and characterisation of these long-term stem cell cultures hold promise for future models of human placentation and trophoblast development.

More recently, Hori and colleagues developed an STB organoid barrier model with apicobasal polarity reflective of placental architecture [84]. To subvert the inside-out differentiation effect of an ECM, TSCs were cultured in suspension without Matrigel. The resultant organoids had an external cell layer of STBs expressing syndecan 1 (SDC1) and β-human chorionic gonadotropin (β-hCG), with microvilli structures representative of human chorionic villi in vivo. This barrier model is a promising advancement in understanding cellular interactions at the fetal-maternal interface and testing placental permeability during drug development.

#### Organoids Incorporating Vascularisation

Given that the placenta is a highly vascularised organ [22], a limitation of many organoid models is the lack of incorporated vascular networks. Interestingly, Cui and colleagues recently generated trophoblast organoids with villous-like structures, which contained endogenous vascular cells from human-induced pluripotent stem cells (hiPSCs) and resembled features of a first-trimester human placenta [85]. In this protocol, hiPSCs were seeded onto micropillar chips and treated with bone morphogenetic protein 4 (BMP4) then embedded in Matrigel, causing self-assembly of a 3D structure. The resultant placenta-like tissue comprised both trophoblast and vascular lineages: CDX2+ villous CTBs; ENDOU+ STBs; HLA-G+ EVTs; CD31+ endothelial cells; and PDGFB+ pericytes. This was confirmed by scRNAseq, quantitative real-time reverse transcription polymerase chain reaction, immunofluorescence, and flow cytometry analysis. This hiPSC 3D culture model creates the potential to study the early-pregnancy relationship between trophoblast cells and vascular cells in a vascular-like network that includes both lineages.

#### Organoids Incorporating Immune Cells

Recapitulating specific interactions between immune cells and trophoblasts has been the focus of many 3D models. In one recent study, self-renewing EVT organoids derived from first-trimester human placental villi were co-cultured with decidual NK cells to study their dynamic interactions [86]. Decidual NK-EVT interacting pairs were identified using CellPhoneDB, a public archive of receptors, ligands and their interactions, allowing for the analysis of cell-cell communication molecules [86, 87]. In another study, Li and colleagues investigated how exposure to unique uNK cell-derived cytokines influences EVT function using trophoblast organoids [68]. Organoids were exposed to uNK cytokines, including colony stimulating factors 1 and 2, lymphotactin and RANTES. There were 31 downregulated EVT genes which were linked to cell cycle control, aligning with the non-proliferative characteristics of EVTs. On the other hand, 44 upregulated genes were associated with biological processes important for placentation such as epithelial-mesenchymal transition, cell invasion, and cell fusion. These findings elucidate how uNK-trophoblast immunological interactions enhance pregnancy success — in particular, the role of uNK cell cytokines in supporting EVT differentiation and moderating innate and adaptive immune responses during placentation.

### Microfluidic Platforms to Create Placenta-on-a-Chip and Organoid-on-a-Chip Models

Microfluidic organ-on-a-chip and organoid-on-a-chip technologies have emerged as powerful tools for modelling the complex placental environment in vitro. These biomimetic microsystems allow for the precise control of fluid flow and interactions between different cell types, providing a more dynamic and physiologically relevant representation of the native tissue/organ microenvironment. These 3D precision-engineered microfluidics platforms can be easily adapted to apply shear stress, which can induce endothelial cell polarisation and more accurately simulate in vivo conditions [88, 89]. By applying controlled fluid shear stresses, these models can examine the impact of mechanical cues on trophoblast invasion, vascular remodelling, and transport [90].

Microfluidic models of the placenta have used various cellular combinations and configurations, providing the complexity needed to develop a physiologically relevant model system of human tissue. Most of these placental microfluidic models are designed to study placental barrier function, transport and infection, whereas only a few have been used to study maternal-fetal interactions through assessing trophoblast invasion and vascular remodelling. Some trophoblast invasion assays such as traditional transwell assays are simplistic, with inaccurate and tedious analysis, and no control of the cytokine gradient [2]. Adaptations such as xCELLigence^®^ improve upon this by providing a label-free method to monitor cell migration kinetics, though it is expensive. Microfluidic invasion assays have distinct benefits. For example, they can incorporate multiple compartments to reflect different tissue regions, may require fewer cells, allow for real-time monitoring of cellular migration and invasion, and can incorporate controlled cytokine and pressure gradients. Advances in chip design and incorporation of endothelial networks for intravascular perfusion have also enabled precise control over fluxes, allowing for the reproduction of in vivo fluxes seen in human blood vessels. This approach is crucial for long-term culture and has allowed for up to 30 days monitoring on-a-chip [91]. In parallel, organoid-on-a-chip models have emerged, using pluripotent stem cell-derived trophoblasts to generate self-organising 3D structures that recapitulate the placental architecture [92]. These advanced models can now be coupled with other organ systems, such as the endometrium, to study the complex maternal-fetal interactions that are critical to successful placentation and pregnancy outcomes [89, 93, 94]. Together, these platforms represent a significant advancement in placental research, allowing for more comprehensive and physiologically relevant investigations of placental development, function, and pathologies.

#### Models of Implantation

Microfluidic technologies have been implemented to explore the interactions between NK cell subtypes and EVTs. NK cells are the most abundant lymphocytes in the uterus at the maternal-fetal interface during pregnancy and as a result are frequently utilised in 3D placental models [95]. Previous work has described the development of a microfluidic device to model migration, specifically the impact of uNK cells on directed migratory patterns of primary EVTs [96]. A gradient of granulocyte-macrophage colony-stimulating factor (GM-CSF), a cytokine produced by activated uNK cells, was created in side channels, while the central hydrogel channel contained EVTs embedded in Matrigel. Tracking and quantification methods revealed increased motility and direction of EVT invasion in the presence of GM-CSF, suggesting that it plays a necessary role in trophoblast invasion. To assess whether EVTs in in vitro models accurately reflect behaviours in vivo, an implantation-on-a-chip quadruple culture model was utilised to examine the effect of pre-implantation uNK cells on the direction of primary EVT and HTR-8/SVneo cell migration into the maternal uterus [97]. In this model, the central hydrogel channel contained both uNK and DSCs within an ECM of equal parts containing Growth Factor-Reduced Matrigel and Type I collagen. DSCs have previously been shown to regulate immune activity and vascular remodelling of the uterus and inhibit EVT invasion [5, 98]. Here, EVTs displayed considerably decreased ECM invasion compared to when primary human endometrial microvascular endothelial cells were present with or without uNK models. However, EVT invasion in this model was still greater compared to when the ECM hydrogel contained DSCs only [97]. This demonstrated that both DSCs inhibitory and uNK stimulating effects are in action, although DSCs seem more prominent, leading to an overall reduction in EVT invasion. This implantation model incorporates different cell types (epithelial, endothelial, mesenchymal and immune) allowing for control over the simulated placental microenvironment and the recapitulation of EVT invasion. As abnormal EVT invasion is implicated in pregnancy conditions including preeclampsia and fetal growth restriction, these models of embryo implantation have the potential to develop into predictive preclinical models of human pregnancy complications [97, 99, 100].

#### Models of Impaired Angiogenesis

An imbalance in key angiogenic regulators has been linked to hypertensive disorders of pregnancy [101]. We recently investigated the importance of trophoblasts in remodelling endothelial vasculature and the importance of endothelial cells in trophoblast migration using a microfluidic placental model [102]. Under inflammatory conditions, we investigated trophoblast invasion and migration, and the expression of the anti-angiogenic protein, FK506-binding protein-like (FKBPL), and immunomodulatory galectin-3 (Gal-3). In this model, the central hydrogel channel contained human umbilical vein endothelial cells (HUVECs) embedded in collagen I, and the side channel contained ACH-3P trophoblast cells. Pathophysiological inflammation was mimicked using tumour necrosis factor alpha (TNF-α), an inflammatory factor that is elevated in preeclampsia. Our study demonstrated the impact of inflammation on vascular integrity and trophoblast migration. TNF-α increased the expression of FKBPL and Gal-3, and impaired the formation of a continuous vascular network. This finding was consistent with previously reported reduced CD31 expression seen in samples of preeclamptic placenta and decidua, indicating failed angiogenesis [103]. By reproducing preeclampsia-associated inflammatory conditions and the resulting alterations in FKBPL and Gal-3 expression, trophoblast migration and vascular network formation, the model provides a valuable platform for investigating the mechanisms that contribute to abnormal placentation and vascular dysfunction. Consequently, vascularised placental models have potential for advancing research into pregnancy complications associated with impaired angiogenesis, including preeclampsia, fetal growth restriction and placental insufficiency [104].

#### Models of Placental Barrier Function

In the first reported study to develop a 3D vascularised placental barrier model using primary human trophoblasts, researchers cultured primary CTBs with an underlying fibroblast stroma containing an endothelial network [105]. The resultant in vitro barrier model was found to closely mimic the complexity of the placental barrier, in terms of structure, secreted factors and response to oxygen alterations. Including primary cell types, rather than immortalised cell lines, allows for more physiologically relevant insights into placental transport, inflammation, and other key processes [106, 107]. 3D placental models have also been used to study the impact of common environmental exposures such as titanium dioxide nanoparticles (TiO_2_-NPs) on the placental barrier [108]. In this study, a range of TiO_2_-NP concentrations were added to the maternal side of a placental-barrier-on-a-chip model to mimic short and chronic placental exposure to nanoparticles (NPs). After TiO_2_-NP exposure, BeWo trophoblast cells attracted maternal macrophages, a process associated with impaired placental barrier function. This suggests the potential adverse impact of even low NP exposure. NPs are promising drug carriers in the development of targeted therapies, although their long-term risks in human pregnancy are yet to be fully elucidated.

Microfluidic placental barrier models have also been used to investigate placental transfer of pharmaceutical compounds. A study of placental transport of the gestational diabetes drug, glyburide, recapitulated active transport mechanisms mediated by efflux transporters in vivo that limit fetal exposure to maternally administered drugs [109]. This can predict the safety of drugs based on their ability to cross the maternal-fetal interface, which is important for advancing drug screening in pregnancy. Similar platforms have been used to study the transfer of nanoparticles, drugs and environmental contaminants across the placental barrier, while simultaneously assessing barrier permeability and cellular responses to exposure [110, 111]. Consequently, these microphysiological systems provide valuable tools for investigating maternal-fetal transport and facilitate safety assessments of drugs and environmental contaminants during pregnancy. By improving our understanding of placental transport and barrier dysfunction, these models may also contribute to research into pregnancy complications associated with impaired barrier function, including preeclampsia, fetal growth restriction and placental insufficiency [110, 112].

#### Models of Placental Infection

Until recently, phenotypic and functional characterisation of human placental macrophages had solely used isolated cells [40]. With a rise in 3D models, some groups have utilised microfluidic platforms to characterise the role of macrophages in the context of various diseases. For example, Zhu and colleagues designed a placental barrier-on-a-chip to investigate the response of human macrophages (derived from THP-1) to *Escherichia coli* (*E. coli*) [107]. *E. coli* is one of the predominant organisms found in fetal organs and was used in the chip’s maternal channel to model acute placental inflammation. In this study, HUVECs were seeded in the fetal channel while trophoblasts (BeWo cells) were in the maternal channel. The trophoblast layer was first inoculated with *E. coli*, after which macrophages were introduced. A greater number of macrophages were observed to adhere to the trophoblast layer, suggesting the activation of the maternal innate immune system in the *E. coli*-infected placenta. Another study assessed the response of co-cultured decidualised human endometrial stromal cells and macrophages (THP-1) to microbial threat in pregnancy. Using a single-chamber organ-on-a-chip model, TNF-α secretion by macrophages was found to be inhibited in response to inflammatory mediators produced by lipopolysaccharide stimulation [113]. These placental barrier-on-a-chip models present effective platforms to study infection-related placental inflammation, which can be a key driver of adverse pregnancy outcomes such as abnormal fetal development, pre-term birth and neonatal mortality [107, 113–116].

### 3D Bioprinting

Bioprinting technologies have opened exciting new avenues for manipulating cells and ECM in 3D cell culture models. This technological advance has been particularly beneficial for the development of placental models. Extrusion and inkjet (drop-on-demand or continuous) bioprinting are the most commonly used techniques, and the choice of technique used varies according to the model’s complexity, size and mechanical requirements. Regardless of the technique used, bioprinted constructs are consistent in size, while automated printing accelerates the fabrication process and enables large sample numbers for high-throughput workflows. Bioprinting therefore has a wide range of applications including organ-on-a-chip, organoids, in vitro disease modelling, biomimetic models, post-injury 3D implants/scaffolds, and the bioengineering of whole organs.

Recent advances in bioprinting have focused on incorporating primary human trophoblast cells, endothelial cells, and other supporting cell types (such as stromal cells) to recapitulate the key processes occurring at the placental barrier. The use of biomaterials, such as GelMA hydrogels, has also enabled the biofabrication of more complex 3D placental architectures to study cell-cell interactions in a physiologically relevant context [117, 118]. In fact, GelMA has been efficiently utilised as a 3D biomaterial that it is used in most 3D bioprinted placental models [47]. GelMA’s success is likely due to its excellent tuneability, stability at physiological temperatures, and retention of cell adhesion and degradation sites which allow cell-matrix interactions. Researchers have also utilised extrusion bioprinting to embed BeWo or HTR-8/SVneo cells within spiral or multi-strip GelMA scaffolds to observe invasion responses to epidermal growth factor [117, 118]. Furthermore, a more complex system has been created by incorporating HUVECs into a GelMA matrix containing HTR-8/SVneo cells, within a perfusable bioreactor system [117]. In another study, a barrier co-culture model was created by incorporating BeWo trophoblasts with primary placental fibroblasts and primary human placental endothelial cells, with a bioprinted “Membrick” GelMA membrane as an alternative to transwell membranes. Interestingly, the BeWo trophoblasts in this model were able to form a barrier with the use of the bioprinted construct, in the presence or absence of fibronectin [119].

In our laboratory, we have recently developed a novel 3D bioprinted placental organoid model using ACH-3P and hTSCs and the RASTRUM drop-on-demand platform (Inventia Life Science) to bioprint first trimester trophoblast cells in a PEG-based matrix [70]. Matrix stiffness was 1.1 kPa to mimic the stiffness of the decidua basalis, where placental invasion takes place [120]. This was found to be consequential to the proliferation and differentiation of the trophoblasts, when compared to those manually embedded in Matrigel, which is softer. Bioprinted ACH-3P and TSCs readily formed organoids, though bioprinted TSC organoids were significantly smaller than their Matrigel counterparts (*P* < 0.001). Both bioprinted and Matrigel-embedded organoids were able to differentiate into STB organoids (SDC1+ and β-hCG+) and EVT organoids (HLA-G+) under predefined medium. The addition of fibronectin, laminin and collagen IV adhesion peptides and hyaluronic acid to the matrix also improved TSC organoid number and size. Overall, this study demonstrated the development of a high-throughput and biologically relevant bioprinted organoid model, with ACH-3P organoids as a lower-cost supplement to hTSCs. The model’s complexity can be advanced by incorporating varied cell types involved in maternal-fetal interactions and tissue-specific ECM materials. The bioprinted model can be readily modified to mimic changes seen throughout gestation, or in pregnancy disorders such as preeclampsia, by modifying the matrix formulation, stiffness and/or adjusting the oxygen culturing conditions.

## Conclusions and Areas for Further Study

Accurate models of placental tissue have been lacking to facilitate the development of diagnostic tests, treatments and monitoring strategies for preeclampsia and fetal growth restriction, to help prevent stillbirth, preterm birth and other pregnancy complications. Fortunately, researchers have now successfully developed reliable methodologies for creating 3D placental models which hold significant benefits over animal and 2D cell models. This review has shown that, despite these substantial advances, 3D placental models remain limited by incomplete multicellular interactions, variability in matrix selection, limited modelling of gestational-stage-specific placental development, and a lack of standardised criteria for validation. This is particularly important because the placenta undergoes profound structural, cellular and functional changes throughout gestation. As a result, many existing models represent only a narrow developmental window and may not fully recapitulate gestational-age-specific features of human placental biology. 3D models can preserve profiling patterns of NK cells, macrophages, T cells, and antigen-presenting cells [121]. However, substantial gaps remain in incorporating these cells, limiting our investigation into their specific mechanisms and roles within the placenta. There is also the need for a reliable source of EVTs to study invasion and remodelling as the invasive EVT phenotype is unique and difficult to replicate with models, given the lack of access to early pregnancy EVTs or cell lines replicating EVT function [2]. In addition, despite significant progress in the field of placental vascular biology, there is a limited understanding of the regulation of placental microvascular endothelial cells during placental development, due to a lack of vascularised placental models. Establishing clearly defined, tuneable matrices will also help ascertain the role of ECM in normal and abnormal placental development.

Furthermore, as placental modelling technologies continue to advance, efforts to develop standardised and reproducible methodologies across laboratories will become increasingly important, to compare findings and facilitate clinical translation and regulatory acceptance. However, standardisation and validation remain particularly challenging in placental research because no single model can yet adequately reproduce all aspects of placental structure and function. Rather, different 3D model systems possess distinct strengths, and model selection is often driven by the specific biological process being investigated, including trophoblast invasion, vascular development, maternal-fetal transport, immune interactions or endocrine function. Future progress in the field will therefore require balancing the need for harmonised methodologies with the flexibility needed to develop fit-for-purpose models that capture complementary aspects of placental biology.

Whilst the applications of translational 3D in vitro models to studies of preeclampsia and placental function are clearly promising, future research addressing our knowledge gaps is crucial for advancing our understanding of cellular interactions in placental health that in turn will further elucidate the molecular mechanisms involved in both physiological and pathological pregnancy.

## Data Availability

No datasets were generated or analysed during the current study.
